# Primary membranoproliferative glomerulonephritis in Sfax, Tunisia: epidemiologic profile and prognostic factors

**DOI:** 10.11604/pamj.2021.38.218.21059

**Published:** 2021-02-25

**Authors:** Ikram Agrebi, Khawla Kammoun, Najla Dammak, Jamil Hachicha, Tahya Boudawara, Faiçal Jarraya, Mohamed Ben Hmida

**Affiliations:** 1 Nephrology Department, Hedi Chaker University Hospital, Faculty of Medicine of Sfax, Sfax, Tunisia,; 2 Laboratory of Research of Renal Pathology LR 19ES11, Faculty of Medicine of Sfax, Sfax, Tunisia,; 3 Laboratory of Anatomopathology, Habib Bourguiba University Hospital, Faculty of Medicine of Sfax, Sfax, Tunisia

**Keywords:** Membranoproliferative glomerulonephritis, epidemiology, prognosis

## Abstract

**Introduction:**

membranoproliferative glomerulo nephritis (MPGN) is a rare kidney disease with a poor prognosis as 50% of patients attend the end stage renal failure after 10 years of follow up. Several factors have been described associated with poor renal prognosis. The aim of our study is to determine the epidemiologic profile and to identify prognostic factors of MPGN.

**Methods:**

our study is retrospective over a period of 16 years (January 1996 - December 2011) including all cases of primary MPGN aged more than 15 years, collected at the nephrology department of Hedi Chaker University Hospital, Sfax, Tunisia.

**Results:**

we collected 118 cases of primary MPGN, with mean age of 45 (SD 19) years. The incidence of MPGN has decreased from 10 cases/year between 1996 and 1999 to 5 cases/year between 2008 and 2011. Seventy-nine percent of patients (n=93) had renal failure at the moment of diagnosis (e-GFR less than 60 ml/min/1.73m^2^;). After a mean follow-up of 51.9 (SD 44) months, progression to end stage renal failure was observed in 43.5% of followed cases (n=20). On univariate analysis, factors associated with death or progression to end stage renal failure were initial renal failure and sclerotic glomeruli (respectively p at 0.040 and 0.032). Multivariate analysis indicated that initial renal failure was significantly correlated with death or progression to end stage renal failure (HR: 0.14, 95% CI (0.033-0.593), p=0.008).

**Conclusion:**

there has been a decline in the number of cases of MPGN diagnosed in our hospital. The presence of renal failure at diagnosis was associated with death or progression to end stage renal failure.

## Introduction

Membranoproliferative glomerulonephritis (MPGN) is a rare kidney disease that affects children and young adults. Its name is derived from the histologic changes including mesangial hypercellularity and thickening of the glomerular basement membrane [1]. It represents 7 to 10% from all glomerular diseases [2]. Its incidence has decreased considerably over the past few decades in developed countries. This decline could be explained by the decrease in chronic infections. But MPGN is still common in low- and middle-income countries [3]. Traditionally, MPGN was classified according to electronic microscopical findings into 3 types. Recently with the progress in understanding the pathophysiology of MPGN, a new classification was proposed by Sethi *et al*. [4] based on immunofluorescence staining. This classification divides MPGN into immune complex mediated and complement mediated MPGN. MPGN is known to have a poor prognosis since it frequently progresses to end stage renal failure (ESRF). Several prognostic factors have been described in literature such as initial renal failure [5], hypertension [5], presence of crescents and interstitial fibrosis [6]. The impact of the new classification on the prognosis is still not known. The aims of this study were to determine characteristics of MPGN in our population and to identify prognostic factors.

## Methods

**Study design, setting and population:** we carried out a retrospective study from January 1996 until December 2011 in the nephrology department of Hedi Chaker university Hospital, Sfax, Tunisia. The study included all patients with biopsy proven MPGN and aged more than 15 years. Patients with secondary MPGN (hepatitis, chronic infection, auto-immune disease, malignancy) were excluded.

**Data collection:** clinical and laboratory data were collected including the following parameters: age, gender, blood pressure, urine examination, serum creatinine, estimated glomerular filtration rate (eGFR) calculated by the MDRD equation, hemoglobin, C3 and C4 complement fractions, CH50, 24 h proteinuria level.

**Histologic and immunofluorescence studies:** kidney biopsies were processed by conventional light microscopy and immunofluorescence. Biopsy specimens were collected and fixed in Dubosq-Brazil for 4h. They were embedded in alcohol 70° for 16h, alcohol 90° for 1 h and absolute alcohol for 1 h. Then they were paraffin embedded and 2-3μm thick sections were cut. The sections were dried at 60°C for 12 h, and then stained with hematoxylin-eosin, periodic acid Schiff (PAS), trichrome light green (Masson) and silver impregnation. For the immunofluorescence study, cryostat sections (2μm thick) were cut (cryostat SLEE). Sections were examined with antisera to human IgG, IgA, IgM, complement (C3 and C1q), kappa and lambda. Electron microscopic evaluation was not available. Light microscopic diagnosis was confirmed in all cases in the presence of diffuse mesangial hypercellularity and endocapillary proliferation [7]. Kidney biopsies were re-evaluated. MPGN was reclassified using the new classification proposed by Sethi *et al*. [4], according to the immunofluorescence staining, into immune complex mediated MPGN and C3 glomerulopathy. The cases showing immunoglobulin as well as complement staining were classified as immune complex mediated MPGN while cases showing exclusive C3 complement staining were classified as C3 glomerulopathy.

**End point:** follow-up parameter was death or end stage renal failure defined as chronic renal failure requiring chronic dialysis treatment or renal transplantation. Patients without adequate follow-up data were excluded from outcome analyses.

**Statistical analysis:** statistical analysis was performed with SPSS version 20.0. Categorical variables were described as the percentage (frequency). Continuous variables were summarized as the mean (standard deviation) or the median (interquartile range [IQR]) as appropriate. Comparison between groups was performed by chi-square test for categorical variables and one-way ANOVA for continuous variables, or non-parametric tests as appropriate. To study prognostic factors, the end point of this study was death or ESRF. Renal survival probabilities were determined using the Kaplan-Meier method and group comparisons for survival were performed using the log-rank test. Variables with p<0.2 in the univariate analysis were further analyzed with logistic regression analysis. All the variables´ hazard ratios (HR) and corresponding 95% CI were calculated at the same time. The level of significance considered was p <0.05.

## Results

**Figure 1:**
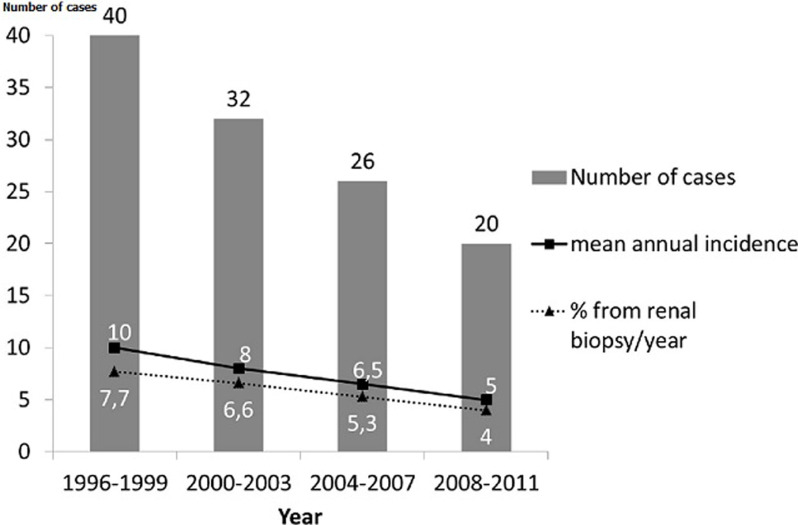
distribution of cases of membrano proliferative glomerulonephritis according to periods of 4 years

**Incidence:** one hundred eighteen cases of idiopathic MPGN were identified. Mean incidence was 7.3 cases/year and 6.2% from all renal biopsy/year. The study of distribution of 4-years groups of patients showed a decrease in the incidence from 10 cases/years between 1996 and 1999, to 5 cases/years between 2008 and 2011 (Figure 1).

**Clinical and laboratory features:** demographic and clinico-biological features of patients at baseline are shown in Table 1. The mean age was 45 (SD 19) years with 65.3% men (n=77). Thirty-nine patients (33%) had a recent history of infection which was in 29.6% (n=35) an upper respiratory tract infection. At presentation, 50.8% of patients (n=60) had nephrotic syndrome. Microscopic hematuria was observed in 75.5% of cases (n=74), hypertension in 73.7% (n=87) and renal failure in 78.8% (n=93) with an average eGFR of 41 (SD 29) ml/min.

**Table 1:** clinico-biological features of membranoproliferative glomerulonephritis at baseline

Variable	Number (%) or mean (SD) or median (IQR)
**Age (years)**	45 (19)
**Gender**	
Male	77(65.3)
Female	41 (34.7)
**Edema**	94 (79.7)
**Hypertension**	87 (73.7)
**Oliguria**	7 (6)
**Hematuria**	74 (75.5)
**Mean Proteinuria (g/24h)**	4.61 (3.17)
**Median Serum Creatinine (µmol/l)**	163.5 (46-1850)
**Mean eGFR (ml/min)**	41 (29)
**eGFR < 60 ml/min**	93 (78.8)
**Clinical presentation**	
Nephrotic syndrome	60(50.8)
Renal failure	24(20.3)
Acute nephritic syndrome	20(16.9)
Hematuria	11(9.3)
Rapidly progressive glomerulonephritis	3 (2.5)
**Mean Serum Albumin (g/l)**	27.75 (6.57)
**Mean Hemoglobin (g/dl)**	10.59 (2.56)
**Anemia**	88 (74.6)
**CRP (ng/ml)**	6.2(0-189)
**Median C3 (g/l)**	0.77(0.17-2.51)
**Low C3**	23 (40.4)
**Median C4 (g/l)**	0.26(0.1-1.5)
**Low C4**	11 (19.3)
**Median CH50 (UI /ml)**	34(34-101)

SD=standard deviation, IQR=Interquartile range, eGFR=estimated glomerular filtration rate, CRP= C-reactive protein

**Histological findings:** histological findings are summarized in Table 2. Crescentic glomerulonephritis was seen in 4.23% (n=5). Interstitial fibrosis was observed in 50.8% (n=60). We found Ribbon-like deposits along the glomerular basement membrane in only 1.7% (n=2). Direct immunofluorescence study of 67 biopsies revealed immune complex mediated MPGN in 59% (n=39) and C3 glomerulopathy in 41% (n=28). Comparison between the two groups showed no statistically significant difference concerning age, gender, clinical presentation and histological findings. Only low serum C3 was more frequent in the group of C3 glomerulopathy (p: 0.056) (Table 3).

**Table 2:** histological parameters of patients with membranoproliferative glomerulonephritis

Variable	Number of case (%)
Influx of neutrophils in glomeruli	44 (37.3)
Crescents	30 (25.4)
Crescentic glomerulonephritis (Crescents > 50%)	5 (4.23)
Lobular appearance of glomeruli	52 (44.1)
Ribbon-like deposits along the glomerular basement membrane	2 (1.7)
Interstitial fibrosis	60 (50.8)
Mild interstitial fibrosis	33 (28)
Moderate interstitial fibrosis	19 (16.1)
Severe interstitial fibrosis	8 (6.8)
Tubular atrophy	39(33.1)
Endarteritis	18 (15.3)
Hyaline arteriosclerosis	16 (13.6)

**Table 3:** comparison between C3 glomerulopathy and immune complex mediated membrano proliferative glomerulonephritis

Variable	C3 glomerulopathy (%) n= 39	immune complex mediated MPGN (%) n= 28	P
**Mean age (SD) years**	41 (17)	48(19)	0.147
**Gender**			
Male	16 (57.1)	27 (69.2)	0.309
Female	12 (42.9)	12 (30.8)	
**Hypertension**	20 (71.4)	30 (76.9)	0.692
**Acute nephritic syndrome**	3 (10.7)	8 (20.5)	0.234
**Nephrotic syndrome**	16 (57.1)	19 (48.7)	0.496
**Renal failure**	19 (67.9)	33 (84.6)	0.105
**Serum Creatinine (median (IQR) µmol/l)**	149.5(49-1620)	177(46-1850)	0.324
**eGFR (mean (SD) ml/min)**	45.39(33.9)	38 (29.8)	0.349
**Proteinuria (mean (SD) g/24h)**	4.7(3.7)	5(3.1)	0.684
**Hematuria**	18 (64.2)	32 (82)	0.554
**Anemia**	22 (78.5)	32 (82)	0.586
**Hemoglobin (mean (SD) g/dl)**	10.6 (3)	10.5 (2)	0.845
**C3 (mean (SD) g/l)**	0.57 (0.4)	0.8 (0.5)	0.227
**Low C3**	8 (28.5)	6 (15.3)	0.056
**C4 (mean (SD) g/l)**	0.27 (0.1)	1.1 (3.6)	0.428
**Low C4**	2 (16.7)	4 (21.1)	0.763
**Percentage of sclerotic glomeruli % (SD)**	21 (31)	8 (13)	0.390
**Crescents**	7 (25)	12 (30.7)	0.771
**Interstitial fibrosis**	15 (53.6)	22 (56.4)	0.813
**Vascular lesions**	7 (25)	9 (23)	0.856

MPGN: membranoproliferative glomerulonephritis, SD=standard deviation, IQR: interquartile range, eGFR=estimated glomerular filtration rate

**Therapeutic attitude:** forty patients (33.9%) were treated with ACE inhibitors or angiotensin II blockers (renin-angiotensin aldosterone system (RAAS) blockade). Fifty-one patients (43.2%) received an antiplatelet therapy. We observed a significant increase in the prescription of RAAS blockade which increased from only 5% (n=2) between 1996 and 2000 to 55% (n=11) between 2008 and 2011 (p < 0.001) against a decrease of antiplatelet therapy prescription. Immunosuppressive agents (steroids with cyclophosphamide) were used in 4 cases with florid crescentic glomerulonephritis (3.4%).

**Figure 2:**
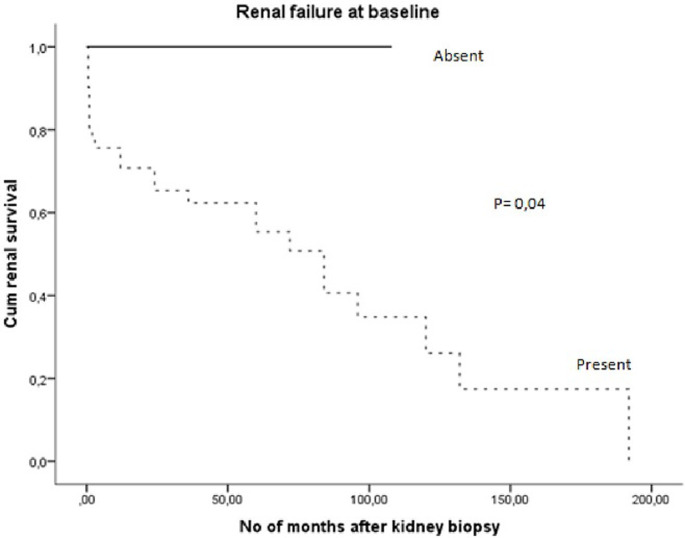
Kaplan-Meier survival plot illustrating the effect of presence of renal failure at baseline on renal survival; renal survival was defined as the progression to end stage renal failure or death

**Renal outcome and prognostic factors:** analysis of outcome included 47 patients with available follow-up data. Mean follow-up period was 51.9 (SD 44) months. Median survival was 84 (56-111) months. Of the 47 followed patients, 15.2% (n=7) had improved their renal function. Death occurred in 10.6% (n=5) and 43.5% (n=20) attended the ESRF within a median period of 12 (0.5-84) months. We found that 34% of patients (n=16) attended the ESRF within 5 years of follow-up. Among patients reaching the ESRF, 4.2% (n=2) underwent renal transplant. Biopsy proven recurrence was observed in the two cases after 48 and 84 months. Using Kaplan-Meier analyses, baseline predictors of death or ESRF during follow-up were presence of renal failure at the time of the renal biopsy (p: 0.040) (Figure 2) and the high percentage of sclerotic glomeruli (p=0.032). On multivariate analysis, presence of renal failure at the time of diagnosis was significantly correlated with progression to ESRF or death (HR: 0.14, 95% CI (0.033-0.593), p=0.008). The significance of each factor affecting the progression to ESRF or death is shown in Table 4.

**Table 4:** impact of clinico-biological findings and histological parameters on renal outcome

	Total (%)	ESRF / Death (%)		Univariable analysis	Multivariable analysis	
		Yes N=24	No N= 23	p value	HR (95% CI)	p value
**Gender**						
Male	28 (59.5)	17 (71)	11 (48)	0.858		
Female	19 (40.5)	7 (29)	12 (52)			
**Age (SD) years**	46.7 (18)	49.8 (18)	43.4 (19)	0.229		
**Age > 60 years**	16 (34)	10 (41.6)	6 (26)	0.177	0.39 (0.082-1.876)	0.243
**Hypertension**	34 (72.3)	20 (83.3)	14 (60.8)	0.506		
**Nephrotic syndrome**	21 (44.7)	11 (45.8)	10(43.4)	0.526		
**Proteinuria > 1g/24h**	41 (87)	23 (96)	18 (78)	0.231		
**Creatinine (median (IQR) µmol/l)**	236	507 (129-1620)	156 (46-1850)	< 0.001	1.00 (0.998-1.002)	0.779
**eGFR < 60 ml/min**	41 (87)	24 (100)	17 (74)	0.040	0.14 (0.033-0.593)	0.008
**Anemia**	38 (81)	19 (79)	19 (82.6)	0.320		
**Low C 3**	9 (19)	4 (16.6)	5 (21.7)	0.813		
**Sclerotic glomeruli (%)**	19.1	25.2	12.7	0.032	1.01 (0.977-1.049)	0.497
**Crescentic glomerulonephritis**	5 (10.6)	3 (12.5)	2 (8.7)	0.091	2.53 (0.308-20.84)	0.386
**Interstitial fibrosis**	28 (59.5)	16 (66.6)	12 (52)	0.393		
**Mild fibrosis**	9 (32)	5 (31.2)	4 (33.3)	0.610		
**Moderate fibrosis**	15 (53.5)	8 (50)	7 (58)	0.746		
**Severe fibrosis**	4 (14.2)	3 (18.7)	1 (8.3)	0.083	0.61 (0.038-9.749)	0.742
**Tubular atrophy**	21 (44.6)	13 (54)	8 (34.7)	0.928		
**Vascular lesions**	16 (34)	11 (45.8)	5 (21.7)	0.376		
**C3 glomerulopathy**	12 (25.5)	7 (29)	5 (21.7)	0.857		

ESRF: end-stage renal failure, HR: hazard ratio, SD: standard deviation, IQR: interquartile range

## Discussion

Our study showed that MPGN is a rare kidney disease as it represents only 6.2% from all renal biopsies/year, with a significant decline in its incidence over recent years in our country. According to literature, MPGN is rare. It represents 7 to 10% from all glomerular diseases [2]. The incidence of MPGN is variable in different parts of the world. It has shown a decline in developed countries like France [8], United Kingdom [9] and USA [10], but still common in low- and middle-income countries due to frequent chronic bacterial and viral infections [3, 11]. The current study showed that the incidence of MPGN had significantly declined over the 16-year period of the study as compared to developed countries. This incidence had also declined when compared with results of the study of Hachicha *et al*. concerning the same country between 1977 and 1990 [12]. Our data are in concordance with the results of previous studies in our country. Ben Maiiz *et al*. reported that the proportion of MPGN decreased from 21.6% between 1975 and 1985 to 13.8% between 1985 and 1995, and only 7.7% between 1995 and 2005 [13]. Different factors could explain this decline such the improvement of the quality of life by the progression of the GDP per capita, the improve of socio-economic conditions [13] and the better control of streptococcal infections by the early use of antibiotic treatment [8, 14].

Consistent with previous reports [5, 9], our study demonstrates that MPGN affected young adults with a mean age of 45 years. The clinical presentation is extremely variable as seen in our study. The most frequent presentation was nephrotic syndrome in 51%. This variability could be explained by the difference in pathogenesis and the timing of the diagnosis. Patients who presented in the early process of the disease are more likely to have nephritic syndrome, and those who had nephrotic presentation are more likely to have sclerotic lesions in kidney biopsy [2].

According to the literature, MPGN is known to have a poor prognosis when compared with other glomerulonephritis. This could be due to the absence of definite target or specific treatment for primary MPGN [1]. Treatment of MPGN is controversial. According to old studies, the treatment of choice was based on antiplatelet therapy with combination of Aspirin and dipyridamole. This therapeutic combination was used since platelets were previously incriminated in the pathogenesis of MPGN [15]. This therapy was abandoned because of lack of benefit and the increase of bleeding risk in case of renal failure [2, 16]. In the late 1990s, RAAS blockade were introduced in the treatment of MPGN based on their antiproteinuric effect [17]. This could explain the evolution of the therapeutic attitude in our center with a decrease in the prescription of antiplatelet therapy since the 2000s against an increase in the prescription of RAAS blockade.

In our study, we confirmed the poor outcome of MPGN as 43% of patients attended the ESRF. Presence of renal failure at diagnosis was a predictor of progression to ESRF or death. This is widely known and has been reported in several previous studies [5, 18-20]. Presence of high percentage of sclerotic glomeruli in kidney biopsy at time of diagnosis was also correlated with progression to ESRF or death. Glomerulosclerosis is an indicator of chronic renal damage and it is known that it is a predictor of evolution to ESRF. Many other risk factors have been reported in literature such as hypertension [5, 20], crescents on renal biopsy [6] and interstitial fibrosis [6, 18-20]. However, in this study, only renal failure was a predictor of outcome. The limits of our study are the retrospective nature of the design and the small number of patients with adequate follow-up.

## Conclusion

There has been a decline in the number of cases of MPGN diagnosed in our hospital. The presence of renal failure at diagnosis was associated with death or progression to end stage renal failure in our population. More prospective studies are needed to confirm these findings.

### What is known about this topic

- MPGN is declining in developed countries;
- MPGN is still common in low- and middle-income countries.

### What this study adds

- The number of cases of MPGN diagnosed in our hospital is also declining;
- Renal failure at the time of initial diagnosis was associated with death or end-stage renal disease requiring dialysis or renal transplantation.
